# Progranulin Is Associated with Disease Activity in Patients with Rheumatoid Arthritis

**DOI:** 10.1155/2015/740357

**Published:** 2015-08-03

**Authors:** Lucie Andrés Cerezo, Markéta Kuklová, Hana Hulejová, Zdeňka Vernerová, Nikola Kaspříková, David Veigl, Karel Pavelka, Jiří Vencovský, Ladislav Šenolt

**Affiliations:** ^1^Institute of Rheumatology, Na Slupi 4, 12850 Prague 2, Czech Republic; ^2^Department of Pathology, Third Faculty of Medicine, Charles University, Šrobárova 50, 100 34 Prague 10, Czech Republic; ^3^Institute of Biophysics and Informatics, First Faculty of Medicine, Charles University, Salmovská 1, 120 00 Prague 2, Czech Republic; ^4^First Orthopaedic Clinic, First Faculty of Medicine, Charles University, V Úvalu 84, 150 06 Prague 5, Czech Republic; ^5^Department of Rheumatology, First Faculty of Medicine, Charles University, Na Slupi 4, 128 50 Prague 2, Czech Republic

## Abstract

*Objective*. Progranulin (PGRN) is implicated in the pathogenesis of rheumatoid arthritis (RA). The aim of this study was to assess the relationship between PGRN and disease activity in RA. *Methods.* PGRN levels were evaluated in patients with RA (*n* = 47) and OA (*n* = 42) and healthy controls (*n* = 41). Immunohistochemical analysis of PGRN in synovial tissues was performed. The association between PGRN and C-reactive protein (CRP), disease activity score (DAS28-CRP), and health assessment questionnaire (HAQ) was studied. *Results*. Circulating PGRN was elevated in patients with RA and OA compared to healthy controls (227.1 ± 100.2 and 221.5 ± 102.5 versus 128.1 ± 34.7 ng/mL; *P* < 0.001). Synovial fluid levels of PGRN were higher in patients with RA compared to OA (384.5 ± 275.3 versus 241.4 ± 165.2 ng/mL; *P* = 0.002). PGRN expression was significantly upregulated in the synovial tissue of RA patients particularly in the inflammatory infiltrates. Serum PGRN levels correlated with DAS28 (*r* = 0.327, *P* = 0.049) and HAQ score (*r* = 0.323, *P* = 0.032), while synovial fluid PGRN correlated only with HAQ (*r* = 0.310, *P* = 0.043) in patients with RA. PGRN levels were not associated with CRP or autoantibodies. *Conclusions*. This study demonstrates increased PGRN expression at local sites of inflammation and association between PGRN levels, disease activity, and functional impairment in patients with RA.

## 1. Introduction

Progranulin (PGRN), also known as granulin epithelin precursor (GEP), PC-cell-derived growth factor (PCDGF), proepithelin, or acrogranin, is an 80 kDa glycoprotein originally identified as an autocrine growth factor for cancer cells and fibroblasts [1]. PGRN is abundantly expressed in rapidly cycling epithelial cells, leukocytes, chondrocytes, and neurons [2] and is involved in biological processes such as embryogenesis [2], tumorigenesis [1, 3] and wound healing [4], inflammation [5, 6], host defense [7], and cartilage development and degradation [8–10].

PGRN also acts as a regulator of the innate immune response and inflammation [5–7]. Under certain conditions, intact PGRN undergoes proteolysis to generate seven 6 kDa peptides, called granulins [6]. PGRN inhibits, whereas granulins stimulate, the production of neutrophil attracting chemokines, which is neutralized by its degradation on granulins by serine proteases NE and PR3 released by neutrophils and macrophages [5, 6]. Mice lacking PGRN respond to infection with exaggerated inflammation and PGRN-deficient macrophages challenged with microbial LPS upregulate the production of proinflammatory cytokines [7].

There is some evidence that PGRN plays a role in systemic autoimmune inflammatory diseases [11–13]. Tang et al. reported that PGRN-deficient mice are susceptible to collagen induced arthritis, and treatment with PGRN can reduce the symptoms of the disease [11]. Similarly, TNF transgenic mice lacking the gene for PGRN developed more severe inflammatory arthritis [11]. It has been demonstrated that PGRN binds to tumor necrosis factor receptors (TNFRs) and thereby limits the action of TNF*α* in inflammatory arthritis [11]. PGRN also potently suppresses cartilage destruction via inhibition of ADAMTS-7/ADAMTS-12-mediated COMP degradation and therefore plays a significant role in preventing the joint destruction in arthritis [8]. Recent study found elevated serum PGRN levels in patients with systemic lupus erythematosus, its association with disease activity, and significant decrease of PGRN serum level after successful treatment [13]. Moreover, Thurner et al. reported the presence of PGRN antibodies that can bind and neutralize PGRN in the sera of patients with rheumatoid arthritis (RA) [14].

Therefore the aim of our study was to assess the PGRN expression in synovial tissue, synovial fluid, and serum in patients with RA and to investigate the relationship between PGRN levels and disease activity.

## 2. Material and Methods

### 2.1. Patients

Forty-seven patients with active RA (35 females and 12 males; mean age ± SD: 58.72 ± 12.05 years), 42 patients with knee OA (26 females, 16 males; mean age ± SD: 64.55 ± 11.12 years), and 41 healthy individuals (30 females, 11 males; mean age ± SD: 56.07 ± 7.35 years) were enrolled in this study. All the patients with RA fulfilled the revised criteria of the American College of Rheumatology (ACR) for the diagnosis of RA [15]. Disease activity of RA was assessed according to the 28-Joint Count Disease Activity Score (DAS28-CRP), which was calculated using the number of swollen and tender joints, CRP levels, and patient global health visual analogue scale (VAS). Health Assessment Questionnaire (HAQ) score was assessed. Characteristics of the patients are given in Table 1. All the patients signed informed consent forms, and the study was approved by the Local Ethics Committee of the Institute of Rheumatology in Prague.

### 2.2. Laboratory Measurements

Blood samples were collected from all the patients when they underwent therapeutic arthrocentesis of the knee or not more than 5 days after arthrocentesis. Paired samples were immediately centrifuged, and both the serum and synovial fluid were stored at −80°C until analysed. Before analysis, the Hylase-Dessau treatment, including heating of the synovial fluids for 30 min at 37°C, was performed. Commercially available ELISA kit (Adipogen Inc., Seoul, Korea) with the detection limit of 32 pg/mL and with the assay range 0.063 ng/mL–4 ng/mL was used to analyse the levels of PGRN in the serum and in the synovial fluid. Absorbance was detected by the Sunrise ELISA reader (Tecan, Salzburg, Austria) with 450 nm as the primary wavelength. CRP levels were determined via an immunoturbidimetric technique using an Olympus biochemical analyser (model AU 400, Japan). Analysis of serum levels of anticitrullinated protein/peptide autoantibodies (ACPA) and IgM rheumatoid factor (IgM-RF) was done with the standard ELISA kits (Test Line s.r.o., Czech Republic).

### 2.3. Immunohistochemistry

Synovial tissue samples were obtained from six patients with RA and seven patients with OA at the time of arthroscopy or open joint surgery (First Orthopaedic Clinic, First Faculty of Medicine, Prague, Czech Republic). Because of restricted access to healthy synovial tissue, OA synovial tissue samples were used as controls for performing immunohistochemistry. Paraffin-embedded sections of synovial tissues were subjected to immunohistochemistry as described elsewhere in more detail [16]. Monoclonal mouse anti-human progranulin antibody (Enzo Life Sciences, Lörrach, Germany) diluted 1 : 100 in ChemMate antibody diluent (Dako, Cytomation, Glostrup, Denmark) was used. Negative control slides were treated with Isotype IgG (Dako, Cytomation) in a dilution of 1 : 1000. All the sections were analysed semiquantitatively using a Nikon Eclipse E600 microscope operated by an experienced pathologist who was blind to clinical data. The analysis included eight to ten random and nonoverlapping fields of synovial tissue. The intensity of PGRN expression was scored on a four-point scale (0–3). In terms of staining intensity, 0 represented lack of positivity, and scores of 1–3 represented weak, moderate, and strong staining intensity, respectively.

### 2.4. Statistical Analysis

Differences in serum and synovial fluid PGRN levels (adjusted for BMI) between the groups were analyzed using a Wilcoxon two-sample test and within the groups by one-sample Wilcoxon test. The relationships among the variables were determined using Spearman correlation coefficients for nonnormal variables. Differences in the expression of PGRN between RA and OA synovial tissue samples were determined using the Jonckheere-Terpstra test. The data were expressed as mean SD unless stated otherwise. *P* values less than 0.05 were considered statistically significant. Statistical Analysis System (SAS) software and R software were used for performing the computations.

## 3. Results

### 3.1. The Expression of PGRN Is Increased in Rheumatoid Arthritis Synovial Tissue

**Figure 1:**
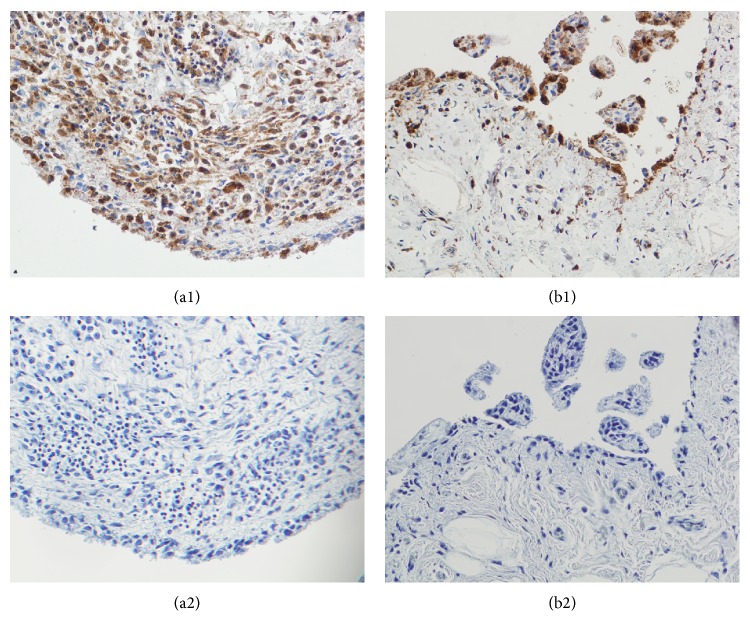
Detection of progranulin (PGRN) protein in rheumatoid arthritis (RA) synovial tissues (a1) and osteoarthritis synovial tissues (b1). Strong staining intensity for PGRN was observed in the lining layer and particularly in the mononuclear cell infiltrates of the sublining layer of RA synovial tissue. Vessels and capillaries were positive for PGRN in both RA and OA synovial tissues. Mouse IgG was used as an isotype control (a2, b2). The original magnification is 200x.

The expression of PGRN was detected in both RA and OA synovial tissues; however, the staining intensity was significantly enhanced in patients with RA (Figure 1). In comparison with OA samples, PGRN was significantly upregulated in the inflammatory cells localized in synovial sublining layer of patients with RA (*P* = 0.033). The synovial lining layer showed slightly higher expression of PGRN in RA compared to OA tissue samples (*P* = 0.086). A comparable PGRN staining intensity was detected in the vessels between the RA and OA synovial tissues (*P* = 0.462) (Table 2).

### 3.2. Synovial Fluid PGRN Levels Are Increased in Rheumatoid Arthritis

**Figure 2:**
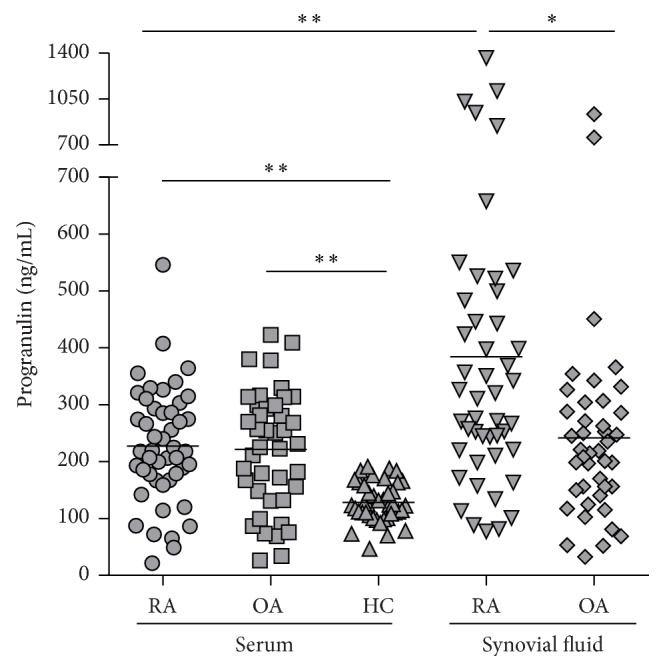
Progranulin (PGRN) levels in the synovial fluid, but not in the serum, were significantly higher in rheumatoid arthritis (RA) compared with osteoarthritis (OA) patients. Serum PGRN was significantly elevated in RA and OA patients compared to healthy controls (HC). The concentration of PGRN in patients with RA was significantly higher in synovial fluid compared to serum. Data are expressed as mean. ^*∗*^ *P* < 0.01, ^*∗∗*^ *P* < 0.001.

The synovial fluid PGRN levels were significantly higher in RA than in OA patients (384.5 ± 275.3 versus 241.4 ± 165.2 ng/mL; *P* = 0.002) (Figure 2). However, circulating PGRN levels did not differ between RA and OA patients, but both were higher compared to those in healthy individuals (227.1 ± 100.2 and 221.5 ± 102.5 versus 128.1 ± 34.7 ng/mL; *P* < 0.001) (Figure 2). The levels of PGRN were significantly elevated in synovial fluid compared to serum in RA patients (*P* < 0.001). In addition, PGRN levels in the serum and in the synovial fluid strongly correlated in RA patients (*r* = 0.551, *P* < 0.0001). There was no correlation between the serum and synovial fluid PGRN observed in OA patients (*r* = 0.205, *P* = 0.199). The levels of PGRN were not affected by sex, age, or treatment.

### 3.3. Associations between PGRN and Disease Activity

**Figure 3:**
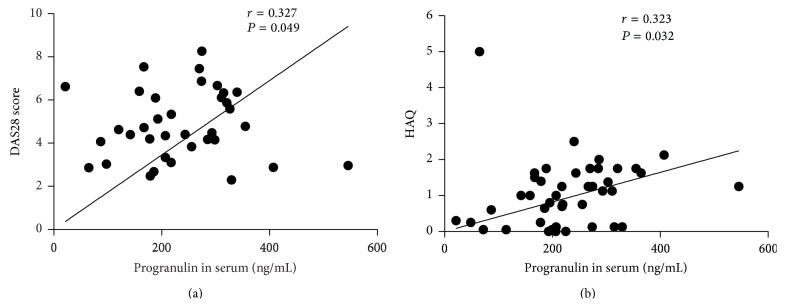
Association of serum PGRN levels with DAS28-CRP (a) and HAQ (b) in patients with rheumatoid arthritis (RA). DAS28: Disease Activity Score; HAQ: Health Assessment Questionnaire score.

Serum PGRN levels correlated with DAS28 (*r* = 0.327, *P* = 0.049) and with HAQ score (*r* = 0.323, *P* = 0.032) (Figures 3(a), 3(b)) in RA patients. However, no relationship between serum PGRN and CRP levels was observed (*r* = 0.126, *P* = 0.403). PGRN in synovial fluid correlated with HAQ (*r* = 0.310, *P* = 0.043), but not with DAS28 (*r* = 0.266, *P* = 0.111). Furthermore, the PGRN levels were not related to the levels of anti-CCP and IgM-RF autoantibodies.

## 4. Discussion

PGRN is implicated in cancer development and is suggested as a growth factor with immunosuppressive properties. In this study we report an upregulation of local PGRN in patients with RA compared with control OA individuals and association between circulating PGRN and RA disease severity, including disease functional impairment.

Animal models have yielded insights into the implication of PGRN in the pathogenesis of RA [11]; however, little is known about its expression in human and its potential association with the clinical and laboratory markers of RA disease activity. The overexpression of PGRN gene in the synovial tissue of RA patients was previously identified by DNA sequencing [17]. In agreement with these data, we show an upregulation of PGRN protein in RA compared to OA synovial tissue with its predominant expression within inflammatory infiltrates of sublining layer. This is in line with PGRN expression by immune cells, particularly macrophages reported earlier [2, 4, 18]. However, it remains to be clarified whether the accumulation of PGRN in RA synovium represents anti- or proinflammatory mechanism modulating the immune response within the joint. In multiple arthritis mouse models PGRN prevented inflammation by inhibition of TNF*α*-activated intracellular signaling and was suggested as an anti-inflammatory molecule [11, 12]. In patients with RA, the levels of serum PGRN correlate with TNF*α* and soluble TNF receptor 2, and the ratio of PGRN/TNF*α* in serum is related to the stage of the disease [18]. This indicates that the role of PGRN in the RA inflammation is rather more complex. Further studies are necessary to elucidate the underlying mechanisms of PGRN, TNF*α*, and TNFR interactions and the role of the PGRN/TNF*α* balance in the pathogenesis of RA. Moreover, it is well established that, during the inflammation, PGRN is cleaved by serine proteases into granulins which are proinflammatory and neutralize the anti-inflammatory effect of progranulin [5]. On the other hand, there is evidence that the full-length progranulin promotes inflammation by upregulating the expression of TNF*α* and IL-1*β* in human monocyte-derived macrophages [19]. Furthermore, PGRN exhibits chemotactic activity for inflammatory cells in cutaneous wound [1]. This indicates that PGRN may play an ambivalent role in control of inflammation depending on the tissue involved.

Consistently with Yamamoto et al. [18], the levels of PGRN were particularly increased at sites of local inflammation compared to the blood circulation of patients with RA. It could be hypothesized that the joint compartment represents a major site of PGRN production in patients with RA. However, as the PGRN autoantibodies were previously detected in the sera of patients with RA [14], it is likely that autoantibodies could bind and thereby neutralize the circulating PGRN in patients with RA. The levels of PGRN in our study were higher in contrast to the study of Yamamoto et al. [18] but comparable to that in patients with systemic lupus erythematosus observed in another study [13]. This may be explained by the use of various antibodies detecting different epitopes in different ELISA assays. In agreement with the previous report [18], the levels of circulating PGRN in both RA and OA patients were comparable and significantly higher in contrast to healthy subjects. Although OA is recognized to involve inflammatory components [20], it is not clear whether this local inflammation may be reflected systemically [21–23]. In addition, PGRN was recently described as a novel chondrogenic growth factor and an OA-related molecule [8–10]. Further studies are therefore needed to elucidate the exact role of the circulating PGRN in patients with OA. Although we show significant local accumulation of PGRN in RA, except functional impairment, neither the disease activity nor CRP levels correlated with the local PGRN levels. However, circulating PGRN levels correlated with disease severity, which has been previously documented also in patients with systemic lupus erythematosus [13] or with CRP levels in obese and type 2 diabetic patients [24]. On the other side, PGRN levels did not correlate with CRP levels in our study but rather reflected global disease activity as well as functional impairment, which might correspond to systemic feature and multiple joint involvement of the disease.

## 5. Conclusions

This study shows significant upregulation of PGRN at local sites of inflammation such as synovial tissue and synovial fluid as well as association between circulating PGRN levels, disease activity, and functional impairment in patients with RA. These findings support further investigation of PGRN in inflammatory diseases, including RA.

## Figures and Tables

**Table 1:** Patients characteristics.

Characteristics	RA	OA	healthy controls
Patients, *n*	47	42	41
Gender (F/M)	35/12	26/16	31/11
Mean age (years)	58.72 ± 12.05	64.55 ± 11.12	56.07 ± 7.35
CRP (mg/L)	24.23 ± 25.78	3.44 ± 3.53	—
Disease duration (years)	6.81 ± 8.14	7.84 ± 8.79	—
DAS28 score	4.55 ± 1.25	—	—
HAQ score	1.06 ± 0.92	—	—
RF positivity, *n* (%)	28 (60%)	—	—
ACPA positivity, *n* (%)	22 (46%)	—	—
DMARDs/GC	42/32	—	—
Biological therapy	9^*^	—	—

ACPA, anti-cyclic citrullinated peptide antibody; CRP, C-reactive protein; DAS28 score, disease activity score; DMARDs, disease-modifying antirheumatic drugs; F, female; GC, glucocorticoids; HAQ score, Health Assessment Questionnaire score; M, male; OA, osteoarthritis; RA, rheumatoid arthritis; RF, rheumatoid factor; SJC, swollen joints count. The data are expressed as the mean (±SD).

^*∗*^Out of 9 patients, 6 were treated with anti-TNF therapy, 1 with tocilizumab, 1 with rituximab and 1 with anti-IL-17 therapy.

**Table 2:** Expression ofprogranulin (PGRN) in different cellular compartments of synovial tissue samples from patients with rheumatoid arthritis (RA) and osteoarthritis (OA).

	RA (*n* = 6)	OA (*n* = 7)	Jonckheere-Terpstra test
Lining layer	2.42 ± 0.48	1.82 ± 0.52	*P* = 0.086
Sublining layer	2.52 ± 0.28	1.68 ± 0.42	*P* = 0.033
Vessels and capillaries	0.31 ± 0.13	0.33 ± 0.10	*P* = 0.462

The intensity of PGRN expression was scored using semiquantitative four-point scale. Score 0 represented no staining, 1 weak staining, 2 moderate staining, and 3 strong staining intensity. The numbers represent mean ± SD.

PGRN: progranulin; RA: rheumatoid arthritis; OA: osteoarthritis.
